# Do pregnancies reduce iron overload in *HFE* hemochromatosis women? results from an observational prospective study

**DOI:** 10.1186/s12884-018-1684-6

**Published:** 2018-02-17

**Authors:** Virginie Scotet, Philippe Saliou, Marianne Uguen, Carine L’Hostis, Marie-Christine Merour, Céline Triponey, Brigitte Chanu, Jean-Baptiste Nousbaum, Gerald Le Gac, Claude Ferec

**Affiliations:** 1 UMR1078 “Génétique, Génomique Fonctionnelle et Biotechnologies”, Inserm, EFS, Université de Brest, ISBAM, 22 avenue Camille Desmoulins, 29200 Brest, France; 2 Laboratoire d’Hygiene et de Sante Publique, Hopital Morvan, Brest, France; 3 Etablissement Français du Sang – Bretagne, Site de Brest, Brest, France; 4 Service d’Hepato-Gastroenterologie, Hopital La Cavale Blanche, Brest, France; 5 Laboratoire de Genetique Moleculaire et d’Histocompatibilite, Hopital Morvan, Brest, France

**Keywords:** Genetic hemochromatosis, Iron overload, Phenotype, Pregnancy

## Abstract

**Background:**

*HFE* hemochromatosis is an inborn error of iron metabolism linked to a defect in the regulation of hepcidin synthesis. This autosomal recessive disease typically manifests later in women than men. Although it is commonly stated that pregnancy is, with menses, one of the factors that offsets iron accumulation in women, no epidemiological study has yet supported this hypothesis. The aim of our study was to evaluate the influence of pregnancy on expression of the predominant *HFE* p.[Cys282Tyr];[Cys282Tyr] genotype.

**Methods:**

One hundred and forty p.Cys282Tyr homozygous women enrolled in a phlebotomy program between 2004 and 2011 at a blood centre in western Brittany (France) were included in the study. After checking whether the disease expression was delayed in women than in men in our study, the association between pregnancy and iron overload was assessed using multivariable regression analysis.

**Results:**

Our study confirms that women with *HFE* hemochromatosis were diagnosed later than men cared for during the same period (52.6 vs. 47.4 y., *P* < 0.001). Compared to no pregnancy, having at least one pregnancy was not associated with lower iron markers. In contrast, the amount of iron removed by phlebotomies appeared significantly higher in women who had at least one pregnancy (e^β^ = 1.50, *P* = 0.047). This relationship disappeared after adjustment for confounding factors (e^β^ = 1.35, *P* = 0.088).

**Conclusions:**

Our study shows that pregnancy status has no impact on iron markers level, and is not in favour of pregnancy being a protective factor in progressive iron accumulation. Our results are consistent with recent experimental data suggesting that the difference in disease expression observed between men and women may be explained by other factors such as hormones.

## Background

*HFE*-related hemochromatosis (or type 1 hemochromatosis; OMIM #235200) is an inborn iron metabolism disorder that is particularly common in Caucasian populations [1, 2]. This genetic disease is characterised by an inappropriate high iron absorption from enterocytes and by an excessive iron release from macrophages. It is due to a defective regulation of the synthesis of hepcidin, the key regulator of iron homeostasis [3–5]. As human body is not capable of eliminating the excess of iron, this will gradually affect various organs and may result in serious damages, e.g. cirrhosis, hepatocellular carcinoma or cardiomyopathy [6].

*HFE* hemochromatosis is inherited as an autosomal recessive trait and is mainly associated with the *HFE* p.[Cys282Tyr];[Cys282Tyr] genotype [7]. The penetrance of this genotype is clearly incomplete [8–10], and its expression is influenced by genetic and environmental factors that may increase or reduce the iron burden [8, 11, 12].

This expression of this genetic disorder is delayed in women than in men. Moreover, *HFE* hemochromatosis women present with a less severe clinical profile, notably a lower prevalence of liver injury [13, 14]. This difference between genders has classically been attributed to the protective effects mediated by iron losses related to menses and pregnancy [15–17].

During pregnancy, maternal iron requirements increase substantially to allow for the physiological expansion of the haemoglobin mass, to promote formation of the foetus and placenta, and to cope with blood losses at delivery [18]. While it is commonly stated in the literature that these pregnancy-associated iron losses are one of the factors that offset the lifelong iron accumulation in women [15–17], this assertion has not been supported by any epidemiological studies in humans.

As recent literature has shown that other factors such as hormones may explain the differences observed according to gender [19, 20], we sought to investigate the association between pregnancy and the phenotypic expression of the predominant *HFE* p.[Cys282Tyr];[Cys282Tyr] genotype.

## Methods

### Study design and participants

This work evaluated a cohort of 140 consecutive p.Cys282Tyr homozygous hemochromatosis women who started phlebotomies between January 1st, 2004 and December 31st, 2011 at a blood centre in western Brittany (Brest, France) where this disease is particularly common [21, 22].

Included patients presented elevated iron markers (with transferrin saturation (TS) > 45% and serum ferritin (SF) > 200 μg/L) and were referred by general practitioners or gastroenterologists to the blood centre for phlebotomy.

### Questionnaire

This study relied on data obtained using a clinical questionnaire that was filled out upon admission to the phlebotomy program. As previously described [22], this questionnaire, which was completed by a referral physician, collected information on socio-demographic characteristics (gender, age at diagnosis, etc.), lifestyle factors (height, weight, alcohol intake, etc.) and biological parameters (including transferrin saturation and serum ferritin).

It also recorded data on reproductive functions, on the presence (and number if any) of pregnancies prior to the beginning of the treatment (excluding miscarriages and abortions occurring in the first trimester), as well as on the menopausal status at admission to the phlebotomy program. Patients were also asked if they were regular blood donors, if they had chronic bleedings (including gastrointestinal bleedings, chronic hematuria, bleedings due to parasitic infections) and if they received blood transfusions (and how many if any), all this prior to admission to the phlebotomy program.

At the end of the depletion stage, treatment-related data (i.e. the number and average volume of the phlebotomies) were recorded. These data enable estimation of the amount of iron removed (AIR; in grams) to normalise patient’s iron stores (i.e. to reach SF < 100 μg/L). This calculation was performed assuming that 1 L of blood contains 0.5 g of iron [23].

### Statistical analysis

Statistical analysis was carried out using the SAS software (version 9.4; SAS Institute Inc., Cary, NC). All tests were performed two-sided, and the significance level was set at 5% for all analyses.

First, we described the baseline characteristics of the studied population. Continuous variables were described in means and standard deviation (SD), and were compared using Student’s *t* test or Anova. When these variables were not normally distributed, they were described by median and interquartile range (IQR), and compared by the Mann-Whitney test. Categorical variables were summarised in percentages, and were compared using χ^2^ test or Fisher’s exact test when appropriate.

Before assessing the influence of pregnancy, we explored the impact of gender on disease expression. For this, we compared the age at diagnosis of women in the study to that observed in the men cared for during the same period in our blood centre (*n* = 161) (using Student’s *t* test). We also evaluated whether the proportion of diagnosed female patients increased with the age at diagnosis i.e. if the sex ratio _(M/F)_ decreased with the age at diagnosis (using a linear trend χ^2^ test).

In a second time, we compared women characteristics according to their pregnancy status (number of previous pregnancies). We then investigated the association between pregnancy and the degree of iron overload (assessed by SF and AIR) using linear regression analysis. As the distributions of these quantitative dependant variables were highly skewed (Kolmogorov-Smirnov test), we performed logarithmic (ln) transformations to normalise them. With such a transformation, the exponential of the estimated regression coefficient (e^β^) indicates how many times the outcome variable varies for each unit increment in the explanatory variable. This means, in other words, that each unit increment in the explanatory variable multiplies the expected value of Y by e^β^. We then tested the association between the iron parameters and potential confounding factors such as age at diagnosis, alcohol intake (whose hepatotoxic effect increases the disease severity) [24, 25]) and menopausal status. We thereafter fitted a multivariable model to enable adjustment for confounders. All explanatory variables associated with the outcome variables at a conservative threshold of *P* < 0.20 in the univariable analysis were included in this multivariable analysis.

In the present study, excessive alcohol intake was defined, in accordance with the World Health Organization definition, as daily consumption exceeding two glasses per day (i.e. 14 glasses per week) for women. Overweight status was defined as a body mass index (BMI) ≥ 25 kg/m^2^.

## Results

### Baseline characteristics of the study population

During the study period, 140 p.Cys282Tyr homozygous hemochromatosis women were enrolled into a phlebotomy program at the blood centre of Brest. The “pregnancy” variable was documented for 137 (97.9%) women and 127 of them (92.7%) completed the depletion phase during the study. The baseline characteristics of the women included in the study and for whom data on pregnancies was available are presented in Table 1. These women were diagnosed in mean at the age of 52.5 years (± 14.0). Among them, 29.8% were overweight (BMI ≥ 25 kg/m^2^) and 4.5% declared having excessive alcohol consumption. More than 60% of the women were menopausal at entry into the phlebotomy program, with a mean age at menopause of 49.2 y. (± 4.3).

**Table 1:** Baseline characteristics of the p.Cys282Tyr homozygous hemochromatosis women according to the number of pregnancies that women had prior to entry into the phlebotomy program

Variables	All women		Number of pregnancies: 0		Number of pregnancies: 1 or 2		Number of pregnancies: ≥ 3		*P* ^*^
	*n*	%	*n*	%	*n*	%	*n*	%	
Number of women	137		14		65		58		
Age at diagnosis *(n = 137)*									
≥ 60 y^a^	41	29.9%	2	14.3%	16	24.6%	23	39.7%	0.077
< 60 y.	96	70.1%	12	85.7%	49	75.4%	35	60.3%	
Body mass index *(n = 131)*									
≥ 25 kg/m^2^	39	29.8%	2	14.3%	19	30.6%	18	32.7%	0.395
< 25 kg/m^2^	92	70.2%	12	85.7%	43	69.4%	37	67.3%	
Alcohol intake *(n = 134)*									
Excessive^b^	6	4.5%	0	0.0%	2	3.1%	4	7.0%	0.418
Non excessive	128	95.5%	13	100.0%	62	96.9%	53	93.0%	
Menopause *(n = 126)*									
Yes	80	63.5%	3	21.4%	36	66.7%	41	70.7%	0.002
No	46	36.5%	11	78.6%	18	33.3%	17	29.3%	
Regular blood donations *(n = 136)*									
Yes	37	27.2%	5	35.7%	15	23.1%	17	29.8%	0.530
No	99	72.8%	9	64.3%	50	76.9%	40	70.2%	
Chronic bleedings *(n = 135)*									
Yes	4	3.0%	2	14.3%	0	0.0%	2	3.4%	0.313
No	131	97.0%	12	85.7%	63	100.0%	56	96.6%	
Blood transfusions *(n = 134)*									
Yes	15	11.2%	1	7.1%	6	9.7%	8	13.8%	0.681
No	119	88.8%	13	92.9%	56	90.3%	50	86.2%	

^a^Classical cut-off for describing the beginning of the expression of *HFE* hemochromatosis in women

^b^Daily consumption ≥2 glasses/day or 14 glasses/week in women (World Health Organization definition)

*χ^2^ or Fisher exact test

### Analysis of the gender difference in expression of the p.[Cys282Tyr];[Cys282Tyr] genotype

Our study confirmed that the age at diagnosis was delayed in women in comparison to the men cared for during the same period (52.6 vs. 47.4 y.; *P* < 0.001). As illustrated in Table 2, the sex ratio decreased significantly with the age at diagnosis, especially after the age of 50 years (χ^2^_linear trend_: 10.5; *P* = 0.001). Thus, women represented about 38% of the p.Cys282Tyr homozygous patients diagnosed before the age of 50 (sex ratio = 1.64), 47.3% of patients diagnosed between 50 and 59 years (sex ratio = 1.12), and 64.6% of patients diagnosed after the age of 60 (sex ratio = 0.55).

**Table 2:** Distribution of men and women by age at diagnosis

Age at diagnosis	Total	Men		Women		Sex ratio
		*n*	%	*n*	%	
< 40 y.	68	42	61.8%	26	38.2%	1.62
[40–50[y.	77	48	62.3%	29	37.7%	1.66
[50–60[y.	91	48	52.7%	43	47.3%	1.12
≥ 60 y.	65	23	35.4%	42	64.6%	0.55
Total	301	161	53.5%	140	46.5%	1.15

### Baseline characteristics of women according to the pregnancy status

The number of pregnancies of the 137 p.Cys282Tyr homozygous hemochromatosis women ranged from zero to six, with an average of 2.4 pregnancies per woman (± 1.3). Nearly 90% of the women in our sample (*n* = 123) had at least one pregnancy before entering the phlebotomy program, with the majority of women having had two (42.3%; *n* = 58) or three (27.0%; *n* = 37) pregnancies. Approximately 15% of the women had four pregnancies or more (15.3%; *n* = 21).

The baseline characteristics and the biological markers of women according to the number of pregnancies (categorised in three groups) are summarised in Tables 1 and 3. As illustrated in Table 1, the age at diagnosis, the proportion of overweight patients and the frequency of alcohol abusers did not differ significantly between the three groups. Similar results were observed for the proportion of patients with previous regular blood donations, chronic bleedings or blood transfusions. At the opposite, a significant association was observed between the number of pregnancies and the proportion of postmenopausal women (*P* = 0.002). Table 3 shows that there was no significant difference in iron markers according to the number of pregnancies.

**Table 3:** Biological parameters of the p.Cys282Tyr homozygous hemochromatosis women according to the number of pregnancies that women had prior to entry into the phlebotomy program

Biological parameters	Number of pregnancies: 0		Number of pregnancies: 1 or 2		Number of pregnancies: ≥ 3		*P*‡
	Median	IQR^a^	Median	IQR	Median	IQR	
Transferrin saturation (%) *(n = 137)*							
	81	[72–89]	78	[65–88]	83	[67–97]	0.998
Serum ferritin (μg/L) *(n = 137)*							
	298	[236–529]	414	[279–693]	412	[297–770]	0.293
Amount of iron removed (g) *(n = 127)*							
	1.3	[1.1–2.0]	2.5	1.4–4.0]	2.3	[1.6–3.6]	0.140
Hemoglobin (g/dL) *(n = 108)*							
	13.6	[13.3–14.6]	14.0	[13.6–14.5]	13.9	[13.4–14.4]	0.815

^a^Interquartile range ([Quartile 1 – Quartile 3])

^‡^Mann-Whitney test

### Association between pregnancy and iron markers

The results of linear regression modelling the association between pregnancy and iron markers (SF and AIR, respectively) are summarised in Tables 4 and 5. Unlike what one might expect, women having had at least one pregnancy did not present lower iron markers than women with no pregnancy. Indeed, the univariable analysis showed that, in comparison to women with no pregnancy, the SF concentration was not different in women who had one or two pregnancies (*P* = 0.288) nor in women who had three or more pregnancies (*P* = 0.126). Combination of these two modalities provided similar findings (e^β^_≥1 vs. 0 pregnancy_ = 1.32; 95% confidence interval [CI]: 0.88–1.97; *P* = 0.177). Adjustment for potential confounders such as age at diagnosis, alcohol intake and menopausal status did not change the observed trends, whatever the coding used.

**Table 4:** Results of the linear regression analysis modelling the association between pregnancies and serum ferritin

Variables	Univariable analysis			Multivariable analysis		
	e β	95% CI	*P*	e β	95% CI	*P*
No. of pregnancies						
0	1.00			1.00		
1 or 2	1.25	[0.82–1.91]	0.288	1.03	[0.67–1.57]	0.902
≥ 3	1.39	[0.91–2.13]	0.126	1.02	[0.66–1.57]	0.944
Age at diagnosis						
< 60 y.^a^	1.00			1.00		
≥ 60 y.	1.35	[1.04–1.73]	0.026	1.20	[0.89–1.62]	0.230
Body mass index						
< 25 kg/m^2^	1.00					
≥ 25 kg/m^2^	1.13	[0.86–1.48]	0.387	–		
Alcohol intake						
Non excessive	1.00			1.00		
Excessive^b^	2.38	[1.34–4.20]	0.003	2.06	[1.11–3.85]	0.023
Menopause						
No	1.00			1.00		
Yes	1.55	[1.20–2.00]	< 0.001	1.39	[1.03–1.87]	0.032
Regular blood donations						
No	1.00					
Yes	0.84	[0.64–1.11]	0.219	–		
Chronic bleedings						
No	1.00					
Yes	0.96	[0.46–1.99]	0.904	–		
Blood transfusions						
No	1.00					
Yes	1.02	[0.69–1.50]	0.938	–		

^a^Classical cut-off for describing the beginning of the expression of *HFE* hemochromatosis in women

^b^Daily consumption ≥ 2 glasses/day or 14 glasses/week in women (World Health Organization definition)

Univariable analysis for pregnancy: global *P*-value = 0.293

**Table 5:** Results of the linear regression analysis modelling the association between pregnancies and the amount of iron removed by phlebotomies

Variables	Univariable analysis			Multivariable analysis		
	e β	95% CI	*P*	e β	95% CI	*P*
No. of pregnancies						
0	1.00			1.00		
1 or 2	1.49	[0.98–2.26]	0.061	1.41	[0.99–2.02]	0.058
≥ 3	1.51	[0.99–2.31]	0.058	1.27	[0.89–1.83]	0.191
Age at diagnosis						
< 60 y.^a^	1.00			1.00		
≥ 60 y.	1.19	[0.90–1.56]	0.214	1.11	[0.86–1.43]	0.415
Body mass index						
< 25 kg/m^2^	1.00					
≥ 25 kg/m^2^	0.96	[0.72–1.27]	0.766	–		
Alcohol intake						
Non excessive	1.00			1.00		
Excessive^b^	2.29	[1.32–3.97]	0.004	1.32	[0.80–2.19]	0.279
Menopause						
No	1.00			1.00		
Yes	1.28	[0.97–1.71]	0.084	0.92	[0.72–1.19]	0.530
Regular blood donations						
No	1.00					
Yes	1.01	[0.76–1.34]	0.930	–		
Chronic bleedings						
No	1.00					
Yes	1.01	[0.50–2.05]	0.979	–		
Blood transfusions						
No	1.00					
Yes	0.98	[0.67–1.43]	0.924	–		
Baseline ferritin	1.00	[1.00–1.00]	< 0.001	1.00	[1.00–1.00]	< 0.001

^a^Classical cut-off for describing the beginning of the expression of *HFE* hemochromatosis in women

^b^Daily consumption ≥2 glasses/day or 14 glasses/week in women (World Health Organization definition)

Univariable analysis for pregnancy: global *P*-value = 0.140

Similar findings were obtained for the second iron marker: AIR (Table 5). The univariable analysis revealed no significant association between pregnancy and AIR. When comparing women who had at least one pregnancy to women with no pregnancy, AIR was even significantly higher (i.e. 1.5 time higher) in women who had at least one pregnancy (e^β^_≥1 vs. 0 pregnancy_ = 1.50; 95% CI: 1.01–2.23, *P* = 0.047). However, this relationship became non-significant after adjustment for potential confounders (e^β^_≥1 vs. 0 pregnancy_ = 1.35; 95% CI: 0.96–1.90, *P* = 0.088). The results remained still at the limit of significance after adjustment for age at diagnosis, alcohol consumption, menopausal status and baseline SF level, when comparing women having one or two pregnancies to women having no pregnancy (*P* = 0.058).

## Discussion

Pregnancy has been suggested to be one potential factor responsible for the later manifestation of *HFE* hemochromatosis in women [15–17]. Yet, our work is the first epidemiological study entirely devoted to the analysis of the association between pregnancy and the phenotypic expression of the main *HFE* genotype in humans. Our study confirms that p.Cys282Tyr homozygous women are diagnosed at a later age than men, and thus corroborates the existence of a difference in the expression of this genotype between men and women. Nevertheless, these results do not confirm the protective effect typically attributed to pregnancy to explain the slower iron accumulation in women.

Our study was subject to little selection bias for several reasons. First, it was a cohort study that included prospectively almost all of the p.Cys282Tyr homozygous hemochromatosis women enrolled in a phlebotomy program at our centre over the study period. Second, the rate of missing values for the main explanatory variable (pregnancy) was very low (~ 2.0%), making our sample fully representative of the p.Cys282Tyr homozygous women who come to medical attention in our area. Finally, we also ensured that the baseline characteristics of women excluded from the multivariable analysis (due to missing values) did not differ from those of included women.

Moreover, iron burden was measured using SF but also AIR, which is the reference method to assess body iron stores [26]. AIR is a more reliable marker than SF, which may also be increased beyond the real degree of iron burden by secondary causes of hyperferritinaemia as excessive alcohol intake or metabolic or inflammatory syndromes [27].

Our study was also able to take into account major confounders, as alcohol intake or menopausal status at entry into the phlebotomy program. Nevertheless, we did not have information on the presence of some other factors susceptible to modify the iron burden (iron supplementation during pregnancy, blood losses from labor and delivery, postpartum hemorrhage, pregnancy complications (pre-eclampsia, abruption, placenta previa), importance of menstrual blood losses, ...) [28]. However, most of these data are not easy to quantify precisely. Some of them may also only have a small effect on iron status, as maternal breastfeeding because its duration (with its consecutive amenorrhea) is in average relatively short and because very little iron is transferred to the milk. We are also aware that it would have been ideal to know the delay between various pregnancies, as well as between the last pregnancy and the beginning of the treatment.

To the best of our knowledge, no epidemiological study has so far been exclusively devoted to the study of the relationship between pregnancy and iron overload in *HFE* hemochromatosis in humans. Some studies have nevertheless shown interest to pregnancy. In a study comparing clinical features of 176 women and 176 matched men [14], Deugnier et al. mentioned that they found no significant correlation between the number of pregnancies and the hepatic iron concentration or the AIR, but no data were presented. They also observed, through a population-based screening study [13], that the number of pregnancies did not differ between 23 expressive and 19 non expressive women. More recently, the same team explored pregnancy as a potential confounder in a model assessing the relationship between body mass index and iron burden in *HFE* hemochromatosis [29]. They found no significant association between the number of pregnancies and AIR (≥ 6 g or < 6 g) in univariable analysis. All these data are consistent with our results.

Our findings seem quite plausible in the current context of fertility. Given the 2016 French fertility rate estimated at 1.93 children per woman [30], it is not surprising that iron losses resulting from an average of two pregnancies per woman are not sufficient to protect against this disease. This situation was most likely different in the past when the fertility rate was higher.

During pregnancy, the daily requirements for absorbed iron markedly increase, from approximately 0.8 mg/day in the first trimester to ~ 8 mg/day in the third trimester [18, 31–33]. Globally, for a singleton pregnancy, a woman needs up to one gram of iron to ensure the balance of iron (depending on iron stores at the beginning of gestation). This corresponds to ~ 500 mg for the physiological expansion of haemoglobin mass, ~ 315 mg for the constitution of foetal tissue and placenta and ~ 250 mg for basal losses [32, 34, 35]. Blood losses at delivery also account to about 150 to 250 mg iron. These additional needs are drawn from the reserves of the mother and are transported to the foetus via the placenta. To cope with extra needs and to replenish the maternal stores, intestinal iron absorption also increases during pregnancy (about approximately 25%) [36]. A part of iron is also made available from the stopping of menses (although this is not sufficient) and from prophylactic iron supplementation that is usually recommended.

It would have been interesting to compare the amount of iron lost during pregnancy to that lost during menses. For example, it has been shown that healthy women with normal menses lose an average of 26 to 65 mL of blood per cycle [37–39], which corresponds at most to a loss of 1 mg iron per cycle (according to the recent assays performed by Napolitano et al.) (20) [38]. Therefore, if we consider that the entire childbearing period lasts average of 40 years (from the mean age at menarche (~ 12 y.) until the mean age at menopause (51 y.)), the total quantity of iron lost due to menses over a lifetime is approximately 520 mg (assuming 13 cycles of 28 days per year). This quantity appears lower than that lost during one pregnancy.

Our findings are also consistent with the results of an experimental study in a mouse model [20] showing that multiple pregnancies do not reduce body iron stores in *Hfe*^−/−^ mice. This study found that all relevant clinical parameters of hemochromatosis (except TS) were not significantly decreased (or even increased) in multiparous females compared with nulliparous females. The hepatic expression of hepcidin [40] and its regulator (BMP6) [41] was reduced in multiparous females, suggesting that the inhibition of intestinal iron absorption was inactivated in response to pregnancy.

Current experimental data suggest that other factors such as hormonal factors may explain the difference in disease expression observed between men and women. Recent findings revealed that the gender difference observed in diseases associated with altered hepcidin expression such as *HFE* hemochromatosis may be explained by the negative regulation of hepcidin transcription by testosterone [19]. Latour et al. showed that testosterone inhibits hepcidin transcription in mice, via enhancement of epidermal growth factor (EGF) receptors signalling in the liver (knowing that EGF was recently shown to inhibit liver hepcidin synthesis [42]). The authors stated that the selective inhibition of EGF receptor in male mice stops testosterone-induced repression and clearly increases hepcidin expression. Moreover, castration of male mice enhances hepcidin expression thus lowers iron overload. Therefore this work highlights that testosterone should be one major hormone responsible for the observed gender difference in regulation of iron metabolism.

## Conclusions

Our work challenges an old and well-established, yet unproven, hypothesis that pregnancy slows iron accumulation in women with *HFE* hemochromatosis. Combined with recent experimental data from the literature, our findings clearly show that the effect of pregnancy is not as important as initially announced and that the search for the factors responsible for the gender difference should continue.

## References

[1] EASL clinical practice guidelines for HFE hemochromatosis. European Association For The Study Of The Liver. J Hepatol. 2010;53:3–22. 10.1016/j.jhep.2010.03.001 20471131

[2] Adams P, Brissot P, Powell LW (2000). EASL international consensus conference on haemochromatosis. J Hepatol.

[3] Anderson GJ, GD ML (2012). Iron physiology and pathophysiology in humans.

[4] Bridle KR, Frazer DM, Wilkins SJ, Dixon JL, Purdie DM, Crawford DH (2003). Disrupted hepcidin regulation in HFE-associated haemochromatosis and the liver as a regulator of body iron homoeostasis. Lancet.

[5] Nemeth E, Tuttle MS, Powelson J, Vaughn MB, Donovan A, Ward DM (2004). Hepcidin regulates cellular iron efflux by binding to ferroportin and inducing its internalization. Science.

[6] Pietrangelo A (2007). Hemochromatosis: an endocrine liver disease. Hepatology.

[7] Feder JN, Gnirke A, Thomas W, Tsuchihashi Z, Ruddy DA, Basava A (1996). A novel MHC class I-like gene is mutated in patients with hereditary haemochromatosis. Nat Genet.

[8] Bacon BR, Britton RS (2008). Clinical penetrance of hereditary hemochromatosis. N Engl J Med.

[9] Beutler E, Felitti VJ, Koziol JA, Ho NJ, Gelbart T (2002). Penetrance of 845G>a (C282Y) HFE hereditary haemochromatosis mutation in the USA. Lancet.

[10] McCune A, Worwood M (2003). Penetrance in hereditary hemochromatosis. Blood.

[11] Allen KJ, Gurrin LC, Constantine CC, Osborne NJ, Delatycki MB, Nicoll AJ (2008). Iron-overload-related disease in HFE hereditary hemochromatosis. N Engl J Med.

[12] Rochette J, Le Gac G, Lassoued K, Ferec C, Robson KJ (2010). Factors influencing disease phenotype and penetrance in HFE haemochromatosis. Hum Genet.

[13] Deugnier Y, Jouanolle AM, Chaperon J, Moirand R, Pithois C, Meyer JF (2002). Gender-specific phenotypic expression and screening strategies in C282Y-linked haemochromatosis: a study of 9396 French people. Br J Haematol.

[14] Moirand R, Adams PC, Bicheler V, Brissot P, Deugnier Y (1997). Clinical features of genetic hemochromatosis in women compared with men. Ann Intern Med.

[15] Bacon BR, Adams PC, Kowdley KV, Powell LW, Tavill AS (2011). Diagnosis and management of hemochromatosis: 2011 practice guideline by the American Association for the Study of Liver Diseases. Hepatology.

[16] Hanson EH, Imperatore G, Burke W (2001). HFE gene and hereditary hemochromatosis: a HuGE review. Human genome epidemiology. Am J Epidemiol.

[17] Wood MJ, Powell LW, Ramm GA (2008). Environmental and genetic modifiers of the progression to fibrosis and cirrhosis in hemochromatosis. Blood.

[18] Milman N (2006). Iron and pregnancy--a delicate balance. Ann Hematol.

[19] Latour C, Kautz L, Besson-Fournier C, Island ML, Canonne-Hergaux F, Loreal O (2014). Testosterone perturbs systemic iron balance through activation of epidermal growth factor receptor signaling in the liver and repression of hepcidin. Hepatology.

[20] Neves JV, Olsson IA, Porto G, Rodrigues PN (2010). Hemochromatosis and pregnancy: iron stores in the Hfe−/− mouse are not reduced by multiple pregnancies. Am J Physiol Gastrointest Liver Physiol.

[21] Jouanolle AM, Fergelot P, Raoul ML, Gandon G, Roussey M, Deugnier Y (1998). Prevalence of the C282Y mutation in Brittany: penetrance of genetic hemochromatosis?. Ann Genet.

[22] Saliou P, Le Gac G, Mercier AY, Chanu B, Gueguen P, Merour MC (2013). Evidence for the high importance of co-morbid factors in HFE C282Y/H63D patients cared by phlebotomies: results from an observational prospective study. PLoS One.

[23] Haskins D, Stevens AR, Finch S, Finch CA (1952). Iron metabolism; iron stores in man as measured by phlebotomy. J Clin Invest.

[24] Fletcher LM, Dixon JL, Purdie DM, Powell LW, Crawford DH (2002). Excess alcohol greatly increases the prevalence of cirrhosis in hereditary hemochromatosis. Gastroenterology.

[25] Scotet V, Merour MC, Mercier AY, Chanu B, Le Faou T, Raguenes O (2003). Hereditary hemochromatosis: effect of excessive alcohol consumption on disease expression in patients homozygous for the C282Y mutation. Am J Epidemiol.

[26] Brissot P, Bourel M, Herry D, Verger JP, Messner M, Beaumont C (1981). Assessment of liver iron content in 271 patients: a reevaluation of direct and indirect methods. Gastroenterology.

[27] Deugnier Y, Bardou-Jacquet E, Le Lan C, Brissot P (2009). Hyperferritinemia not related to hemochromatosis. Gastroenterol Clin Biol.

[28] Blanco-Rojo R, Toxqui L, Lopez-Parra AM, Baeza-Richer C, Perez-Granados AM, Arroyo-Pardo E (2014). Influence of diet, menstruation and genetic factors on iron status: a cross-sectional study in Spanish women of childbearing age. Int J Mol Sci.

[29] Desgrippes R, Laine F, Morcet J, Perrin M, Manet G, Jezequel C (2013). Decreased iron burden in overweight C282Y homozygous women: putative role of increased hepcidin production. Hepatology.

[30] https://www.insee.fr/fr/statistiques/2554860.

[31] Viteri FE (1994). The consequences of iron deficiency and anaemia in pregnancy on maternal health, the foetus and the infant. SCN News.

[32] Leong WILB (2012). Iron nutrition. Iron physiology and pathophysiology in humans.

[33] Cao C, O'Brien KO (2013). Pregnancy and iron homeostasis: an update. Nutr Rev.

[34] Food and Nutrition Board, Institute of Medicine (2002). Dietary reference intakes for vitamin a, vitamin K, arsenic, boron, chromium, copper, iodine, iron, manganese, molybdenum, nickel, silicon, vanadium, and zinc.

[35] FAO/WHO (2004). Requirements of vitamin a, iron, folate and vitamin B12. FAO food and nutrition series. Volume N°23.

[36] Millard KN, Frazer DM, Wilkins SJ, Anderson GJ (2004). Changes in the expression of intestinal iron transport and hepatic regulatory molecules explain the enhanced iron absorption associated with pregnancy in the rat. Gut.

[37] Harvey LJ, Armah CN, Dainty JR, Foxall RJ, John Lewis D, Langford NJ (2005). Impact of menstrual blood loss and diet on iron deficiency among women in the UK. Br J Nutr.

[38] Napolitano M, Dolce A, Celenza G, Grandone E, Perilli MG, Siragusa S (2014). Iron-dependent erythropoiesis in women with excessive menstrual blood losses and women with normal menses. Ann Hematol.

[39] Dasharathy SS, Mumford SL, Pollack AZ, Perkins NJ, Mattison DR, Wactawski-Wende J (2012). Menstrual bleeding patterns among regularly menstruating women. Am J Epidemiol.

[40] Nicolas G, Viatte L, Lou DQ, Bennoun M, Beaumont C, Kahn A (2003). Constitutive hepcidin expression prevents iron overload in a mouse model of hemochromatosis. Nat Genet.

[41] Andriopoulos B, Corradini E, Xia Y, Faasse SA, Chen S, Grgurevic L (2009). BMP6 is a key endogenous regulator of hepcidin expression and iron metabolism. Nat Genet.

[42] Goodnough JB, Ramos E, Nemeth E, Ganz T (2012). Inhibition of hepcidin transcription by growth factors. Hepatology.

